# Improved supervised classification of accelerometry data to distinguish behaviors of soaring birds

**DOI:** 10.1371/journal.pone.0174785

**Published:** 2017-04-12

**Authors:** Maitreyi Sur, Tony Suffredini, Stephen M. Wessells, Peter H. Bloom, Michael Lanzone, Sheldon Blackshire, Srisarguru Sridhar, Todd Katzner

**Affiliations:** 1 Department of Biological Sciences, Boise State University, Boise, Idaho, United States of America; 2 Sky Patrol Abatement, Simi Valley, California, United States of America; 3 U.S. Geological Survey Henderson, Henderson, Nevada, United States of America; 4 Bloom Biological, Santa Ana, California, United States of America; 5 Cellular Tracking Technologies, Rio Grande, New Jersey, United States of America; 6 Department of Computer Science, Boise State University, Boise, Idaho, United States of America; 7 U.S. Geological Survey, Forest and Rangeland Ecosystem Science Center, Boise, Idaho, United States of America; INIBIOMA (Universidad Nacional del Comahue-CONICET), ARGENTINA

## Abstract

Soaring birds can balance the energetic costs of movement by switching between flapping, soaring and gliding flight. Accelerometers can allow quantification of flight behavior and thus a context to interpret these energetic costs. However, models to interpret accelerometry data are still being developed, rarely trained with supervised datasets, and difficult to apply. We collected accelerometry data at 140Hz from a trained golden eagle (*Aquila chrysaetos*) whose flight we recorded with video that we used to characterize behavior. We applied two forms of supervised classifications, random forest (RF) models and K-nearest neighbor (KNN) models. The KNN model was substantially easier to implement than the RF approach but both were highly accurate in classifying basic behaviors such as flapping (85.5% and 83.6% accurate, respectively), soaring (92.8% and 87.6%) and sitting (84.1% and 88.9%) with overall accuracies of 86.6% and 92.3% respectively. More detailed classification schemes, with specific behaviors such as banking and straight flights were well classified only by the KNN model (91.24% accurate; RF = 61.64% accurate). The RF model maintained its accuracy of classifying basic behavior classification accuracy of basic behaviors at sampling frequencies as low as 10Hz, the KNN at sampling frequencies as low as 20Hz. Classification of accelerometer data collected from free ranging birds demonstrated a strong dependence of predicted behavior on the type of classification model used. Our analyses demonstrate the consequence of different approaches to classification of accelerometry data, the potential to optimize classification algorithms with validated flight behaviors to improve classification accuracy, ideal sampling frequencies for different classification algorithms, and a number of ways to improve commonly used analytical techniques and best practices for classification of accelerometry data.

## Introduction

Behavioral constraints can play a substantial role in determining ecological interactions [1], ecosystem processes [2], organismal distributions [3] and animal movements [4]. For flying birds, whose energetic requirements may be substantially greater than those of non-flying animals, costs of locomotion may be so great that they can determine when and how individuals move [5]. Given the great costs birds incur when moving, many species have evolved to specialize on either flapping flight (e.g., hummingbirds, geese; [6,7]) or on soaring and gliding (e.g., oceanic seabirds, vultures; [8,9]). Most species though switch between soaring and flapping flight modes on a fairly regular basis [10] and time spent in these behaviors is a key determinant of the total energetic costs of transport.

Although important to understand flight behavior, it can be difficult to distinguish between avian flight modes using telemetry data [11]. For example, research using GPS telemetry often has described soaring flight in detail, without any reference to flapping flight [8,12]. However, small and portable accelerometry sensors can measure changes in body posture and acceleration to more precisely quantify avian behavior [11,13,14]. These devices provide measures of both static acceleration, that due to the Earth’s gravitational pull, and dynamic acceleration, that due to the movement by the animal. These data are collected in three distinct planes (*x*, *y* and *z* axis) at variable frequencies [15].

The vast majority of published accelerometry studies interpret acceleration data with statistical models developed by experts but without validation to match either modeled predictions with behavioral observations (e.g., [7,8,16,17]) or to automated segmentation and clustering of accelerometer data [18,19]. The few studies that have used supervised classification to create models and validate predicted behaviors have demonstrated dramatic improvements in classification accuracy as a result of validation [9,14].

Golden eagles (*Aquila chrysaetos*) are a large, sometimes migratory raptor that spend much of their flight time soaring and gliding but also engage in steady or intermittent flapping flight [12]. Active flapping flight by golden eagles requires more power than soaring flight [20]. It is therefore reasonable to expect that variation in the amount of time spent in these different flight behaviors is important to the energy budget of individual eagles. Because of the ecological significance of understanding avian flight behavior [12] and the conservation relevance of classifying golden eagle flight in particular [21], there is an important need to improve existing techniques for classification of accelerometry data in general and for this species.

We evaluated the effectiveness of different data collection strategies and two supervised classification systems for classifying accelerometry data to describe flight behaviors of golden eagles as a basis for future behavioral and energetics studies. The questions we asked were: (1) can we compare among and optimize algorithms for classifying accelerometer data of validated flight behaviors to identify mechanisms to improve classification accuracy over previous studies and (2) given these optimized models, what is the ideal accelerometer sampling frequency to use for classification of behaviors? Finally, we demonstrate an application of our classification algorithm by classifying un-validated data collected from wild golden eagles to understand applications and limitations of accelerometry as a tool.

## Methods

The acceleration based behavior classification approach we used required us to relate the statistical properties of the acceleration data to observable behavioral categories of the animal for model training and validation [9, 13, 14]. Our approach had four phases: (1) collection of accelerometry and behavioral observations (validation data), (2) data processing, including matching accelerometry to behavioral observations, (3) statistical modelling and optimizing classification algorithms, and (4) model application. Subsequently, we subsampled our high frequency accelerometer data to identify optimal sampling rates of such data and we applied our final models to classify raw accelerometry data from five wild golden eagles. Each of these steps is detailed below.

### Data collection: Collection of accelerometry and behavioral data

We collected two types of accelerometry data from golden eagles, (1) those from a trained eagle whose flight behaviors we could observe and record on video and (2) those from wild eagles we could not observe. The trained eagle was flown under falconry permits from the State of California to T. Suffredini and with consultation from permitting biologists in USFWS R8 and California Department of Fish and Wildlife. The wild eagles were flown under Animal Care and Use protocols authorized by West Virginia University and were captured and tagged under Federal and State permits to TK.

We outfitted a wild-hatched but rehab captive and trained (by TS) 18-month old golden eagle with a proprietary GPS and tri-axial accelerometry logging device custom designed by Cellular Tracking Technologies, LLC (CTT, Rio Grande, NJ). The GPS–GSM telemetry unit weighed approximately 95 g and was less than 3 percent of body weight of the bird. The device was attached to the bird as a backpack, centered in on the upper third of the spine, with a Teflon ribbon harness (Bally Ribbon Mills, Bally, PA). GPS accuracy and other features not related to the accelerometry system are described elsewhere [22]. When operating, the logger collected accelerometry data at ~140Hz (measurements per second) and stored them in on-board flash memory. Those data were then manually downloaded and parsed as a .csv file.

We flew the trained and outfitted eagle in three different southern California sites that we named “Tehachapi”, “scrub” and “Mojave”, all in habitat types that wild golden eagles are known to use (see S1 Supporting Information for details). A trained videographer (SMW) recorded the flying golden eagle, to the extent possible, with a tripod-mounted or hand held Sony PMW 300 digital video camera. Digital video was processed using XAVC 422 1080p codec and viewed as .mp4 or .mov files.

We also outfitted 5 wild golden eagles in the eastern half of the United States with GPS-GSM telemetry systems with on-board accelerometry (again manufactured by CTT). The attachment system and device hardware were as above, except (a) data collected by these devices were sent via the mobile phone network, rather than manually downloaded; and (b) the accelerometer was programmed only to store data when the bird was in motion, thus saving device memory because no data were collected when the bird was perched.

### Data processing

#### Behavior annotation of the videos

A single observer (MS) annotated all video to create two types of ethograms (Table 1). The first was a simple ethogram based on three basic behavior categories we called flapping, sitting and soaring. For this analysis, we combined soaring, gliding, thermal circling, wing tucks and single wing beats into the flight category “soaring”. Combining these is appropriate because all these behaviors have similar low energetic costs and because combining them simplifies the classification problem (we did attempt to separate out wing tucks [23], but our algorithms were not effective in this regard and so this behavior was grouped with others). Although our main aim was using accelerometry data to describe flight behaviors of golden eagles, we include sitting in our ethograms because of the ecological importance of this behavior. In particular, when and where the birds are sitting provides insight into behaviors associated with defense against extreme weather, foraging opportunities and choice of sites close to environmental updrafts critical for flight behavior of this species.

**Table 1:** Ethogram of behavior used in simple and complex behavioral classification of a trained golden eagle outfitted with an accelerometer.

**Behavior—Simple Ethogram (#1)**	**Behavior definition (as observed in video)**
1. Flapping	At least two flaps of the wings while airborne.
2. Soaring	Gliding, soaring, thermal circling with occasional wing tucks and wing beats.
3. Sitting	Sitting on the ground or mantling.
**Behavior—Complex Ethogram (#2)**	**Behavior definition (as observed in video)**
1. Flapping Straight	At least two wing flaps where body of bird is parallel to the ground.
2. Flapping Banking	At least two wing flaps when body of bird is tilted at an angle of ≳20°.
3. Soaring Straight	Gliding, soaring, thermal circling, wing tucks or wing beats with body parallel to the ground.
4. Soaring Banking	Gliding, soaring, thermal circling, wing tucks or wing beats with body at an angle of ≳20°.
5. Sitting	Sitting on the ground or mantling.

The second ethogram was more complex with five categories that included sitting as well as two subcategories of flapping flight and two subcategories of soaring flight (Table 1). All four subcategories differentiated between banking and straight flights as observed in the video. We use an approximate tilt of 20 degree to different the two, with the angle estimated by the observer classifying video segments. We include these body postures to our ethogram for two reasons. First, the video data of the birds clearly shows these different postures and we wished to test whether they could be identified using accelerometer data as well. Secondly, variations in body postures are indicative of different behavior types and can be used for ecological studies. For example, banking flights in golden eagles are suggestive of searching behavior while straight postures are more common during directional hunting behavior. We also annotated the video when the bird was sitting on the trainers hand and when the bird was outside the video frame. These sections of the video and their associated acceleration data were not used for the classification process but were necessary for precise synchronization of the acceleration data and the video recordings.

#### Segmentation of the accelerometer data

Classification of accelerometry data involves dividing the continuous dataset into either variable- or fixed-time segments to assign boundaries between different behavior classes. Most previous studies have used fixed-time segments, possibly because it involves one less processing step [9,13,24]. However, this approach lowers classification accuracy because the fixed-time boundaries often are not coincident with the boundaries between real behaviors [14].

We used a nonparametric variable-time segmentation method called change point model (CPM) framework that divides the continuous time series of acceleration data into unequal-length segments based on statistical properties of the data (package cpm in R (version 3.2.5); [25,26]). Within the CPM framework, we used a sequential detection technique that allows multiple change points to be detected in a sequence of data points. This modeling technique evaluates the mean and variance at each possible split point using a two-sample test statistic to identify changes in behavior [25].

Preliminary analysis suggested that of the three accelerometer axes, the *x* (sway) signal of the acceleration data was the most responsive to changes in eagle flight behavior. We therefore used *x* axis data to detect change points by implementing the cpm function ‘processStream’. This function requires three parameters: the Average Run Length (ARL_0_), the startup value, and the test statistic to be used (cpmType) [25]. The ARL_0_ parameter specifies the expected number of observations received before a false positive is detected; we set this parameter at 50,000 [14]. The startup value specifies the initial number of observations after which change points are detected; we set this to the average frequency of the acceleration data at each site. For example, in Tehachapi the average number of observations per second was 144 (a frequency of 144Hz), so the startup value was set at 144. Lastly we used a Generalized Likelihood Ratio as our test statistic to detect changes in both mean and variance of the accelerometer data [27].

#### Assigning annotated behavior to segmented accelerometer data

We used the annotated video to assign, for both the simple and complex ethograms (Table 1), behavior categories to the segments of the acceleration data defined by the CPM. In many instances a CPM segment included two, but never more than two, different behavior types. When this happened, we categorized the behavior during that segment as that occupying the majority of time in the segment.

It was not always straightforward to differentiate banking and straight flight from the video recording of the trained bird. This is because the video did not always capture both the bird and the earth’s surface. In cases where the ground was not visible, we estimated the bird’s relative position based on preceding or subsequent frames that included the ground as a reference point.

### Modelling and optimizing classification algorithms

#### Random forest model

For our first model, we used a random forest (RF) algorithm [28] to form a predictive rule for supervised learning and classification of accelerometer data. RF is a 3-step ensemble learning technique that works by: (1) bootstrapping a training data set containing predictor variables, (2) fitting many classification trees to a random selection of predictor variables and (3) combining the predictions from all the trees. Each classification tree recursively partitions the data into binary groups that are increasingly homogeneous to a particular class. When the bootstrapping is done, about 37% of the data are excluded (these are called the ‘out of bag’ data), but the remaining data are replicated to bring the sample to full size. The out of bag data set is used to calculate the classification errors and is an internal cross-validation method in the RF model.

We calculated a list of summary statistics for each segment defined by CPM to evaluate the statistical properties of accelerometer data related to each behavior. We then used these summary statistics as input variables for classifying behavior with the RF algorithm. We calculated separately, for the *x* (sway), *y* (surge) and *z* (heave) signals of the accelerometer data, eight summary statistics similar to those used in previous studies [9,11,14,16,29,30]. These were the mean, minimum value, maximum value, standard deviation, skewness, kurtosis, trend and dominant power spectrum (since there were 3 axes, this resulted in calculation of 24 statistics). In addition we calculated three pair-wise correlations between the *x*, *y*, and *z* axes. Finally, we also calculated the overall dynamic body acceleration (ODBA) which measures the aggregate acceleration of the bird [9, 31] by taking the sum of dynamic body acceleration for each of the three axes. The dynamic body acceleration was derived by first taking a running mean of the raw data for each axis over a window size of 1 sec [32] thereby estimating the static acceleration and then subtracting the static acceleration component from the raw acceleration value for that time period. We used Matlab R2016 (The MathWorks, Inc., Natick, Massachusetts, USA) to calculate kurtosis, skewness and dominant power spectrum values (9 statistics). The remaining 19 statistics were calculated in Microsoft Excel (version, 2013, Microsoft Corporation, Redmond, WA, USA).

We used the ‘randomForest’ package [33] in R (version 3.2.5) to optimize the number of trees grown (ntree; default = 500) and the number of predictor variables that were randomly selected at each node (mtry; default = square root of the total number of predictor variables). In this process, we tested a range of ntree values from 500 to 4000 at intervals of 500, and a range of mtry values from 0 to 28 at unit intervals. We then selected the combination of ntree and mtry values that resulted in the lowest out of bag errors.

We used 70% of the data for training the model (training data set) and we tested the classification accuracy on the remaining 30% of the data (testing data set). This is separate from the “out of bag” error rate noted above. We ran the models once for the simple ethograms and once for the complex ones. We used a confusion matrix to calculate the class-wise estimates of sensitivity, specificity, precision, prevalence and balanced accuracy using the package ‘caret’ [34] in R (S2 Supporting Information). Classification accuracy is based on correctly classified segments. We also estimated the importance of the predictor variables using the function ‘varImpPlot’ within ‘randomForest” package in R which measures the mean decrease in accuracy. The RF model estimates the importance of a variable by measuring the change in prediction error when the out of bag data for that variable are permuted (re-arranged) and all other variables are left unaffected.

Although the 70:30 split is the most common cross-validation method used in accelerometer studies from animal biotelemetry, we also wished to validate our model accuracy with an alternative approach. For this we used cross validation methods for machine learning applications that are commonly seen in human behavior studies [9,35–37]. To do this, we used the full data set in a K-fold cross validation to account for variations in model performance that might be brought about by potential bias in our comparatively small training and test data. A K-fold cross validation works by dividing the data set into K segments. In each run, one segment plays the role of the validation set whereas the other remaining segments (K-1) are the training set. The mean of the classification errors calculated at each run gives the overall cross validation error. We performed ten repetitions of a 10-fold cross validation, using the ‘errorest’ function in the R package ‘ipred’ [38].

#### K-nearest neighbor model

For our second model, we performed a K-nearest neighbor (KNN) classification analysis. KNN is a primitive form of machine learning classification algorithm [38]. It establishes the K-nearest neighbors within a training data set and then classifies each unclassified acceleration datum by identifying multiple most similar (nearest) neighbors from the training data set and using them to infer a class for that data point.

We used the R package ‘class’ [39,40] for the KNN analysis, as follows:

1. We divided the segmented and annotated accelerometry data into a training data set (70% of the data) and a testing data set (30% of the data) based on all acceleration measurements (not based on segments).
2. We randomly mixed the rows of the training data set.
3. We normalized the values of the numeric *x*, *y*, and *z* accelerometer values.
4. We trained the model using the training data set.
5. We estimated the optimal value of K, by using the KNN algorithm to classify the testing data set and then calculating the accuracy (% correct) of classification when the value of K varied from 5 to 50, in increments of 5. Once we identified the two K values with the highest classification accuracy, we then calculated accuracy at unit intervals between those two to isolate the whole number optimal k value.
6. We classified the testing data set (30% of the data) with the KNN algorithm and using the optimal value of K.
7. We used a confusion matrix of the classification results to calculate class-wise estimates of sensitivity, specificity, precision, prevalence and balanced accuracy using the package ‘caret’ in R [34] (SI2).
8. We then performed 10 repetitions of 10-fold cross validation as a second accuracy metric.

#### Optimizing sampling frequencies for accelerometer data

To understand the optimal frequency for collection of accelerometer data from a flying eagle, we subsampled our ~140Hz data to frequencies of 5, 10, 20 and 40 Hz (using Matlab R2016). We then used the two supervised classification algorithms to classify the subsampled data sets with the simple ethogram. Finally, for each subsampled data set, we calculated model classification accuracy with a 70/30 split (as above) and we estimated an inflection point above which model classification accuracy did not improve.

### Application: Classification of data from wild eagles

We applied the RF and KNN classification algorithms to unvalidated accelerometry data from five free-flying golden eagles and we classified them with our simple ethogram. The data from all the birds were grouped together and the two models were then run on this pooled dataset. For consistency, we ensured that the data were collected between 6:00 to 18:00 hours on March 20, 2016. During this time these eagles were migrating and traveling across seven States in eastern North America. The unit configuration and the tag alignment were the same for all the birds (as described above: section data collection). We estimated the proportion of time spent in each behavior by all the birds, and we plotted variation in flight behavior by time of day and by flight altitude.

## Results

We collected a total of 2 hours, 53 minutes and 7 seconds of accelerometer data at ~140 Hz from the trained golden eagle. We also collected a total of 30 min and 21 sec of video in which the bird was visible in the video frame. Of this time, the bird was sitting on the trainer’s glove for 15 min and 11 sec; we annotated the behavior in the remaining 15 min 10 sec of video (S1 Table). The change point model framework identified 1557 distinct behavioral segments that we classified. The average number of data points for all segments, classified and unclassified, was ~ 49. For the simple ethogram the total number of segments in flapping, sitting and soaring behavior was 438, 219 and 900 respectively. The total number of segments in flapping straight, flapping banking, sitting, soaring straight, and soaring banking were 177, 261, 218, 598 and 303 respectively.

### Modelling and optimizing classification algorithms

#### Random forest model

**Fig 1:**
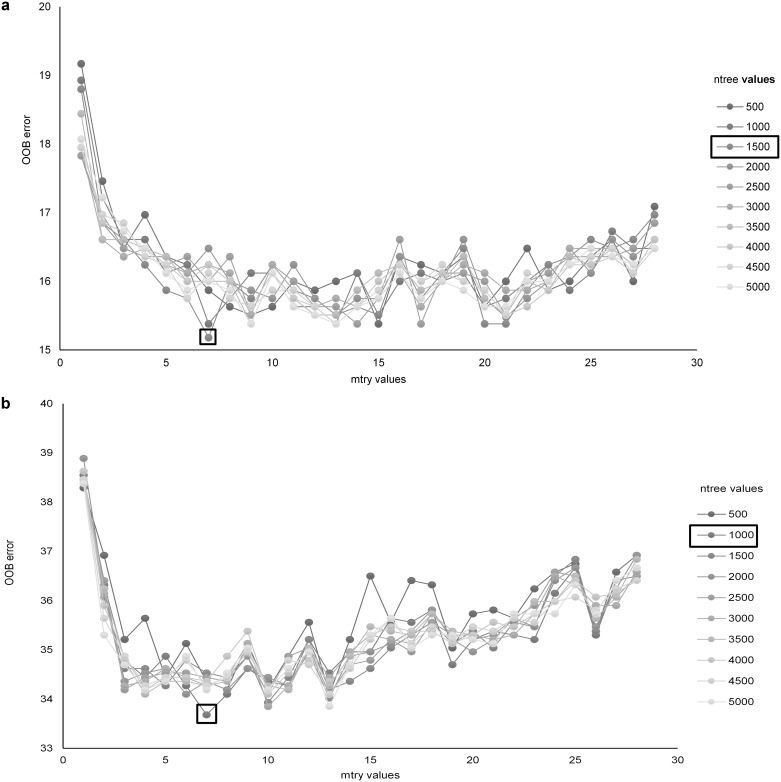
Out of Bag (OOB) errors versus number of predictors, by node, from random forest classification of accelerometer data collected from a trained golden eagle. Number of nodes (mtry) ranged from 0–28 and number of trees (ntree) from 500 to 5000. We classified data to (a) three behavioral classes: flapping, sitting and soaring and (b) five behavioral classes: flapping banking, flapping straight, sitting, soaring banking and soaring straight. Boxes identify combinations of mtry and ntree values resulting in the lowest OOB error.

**Fig 2:**
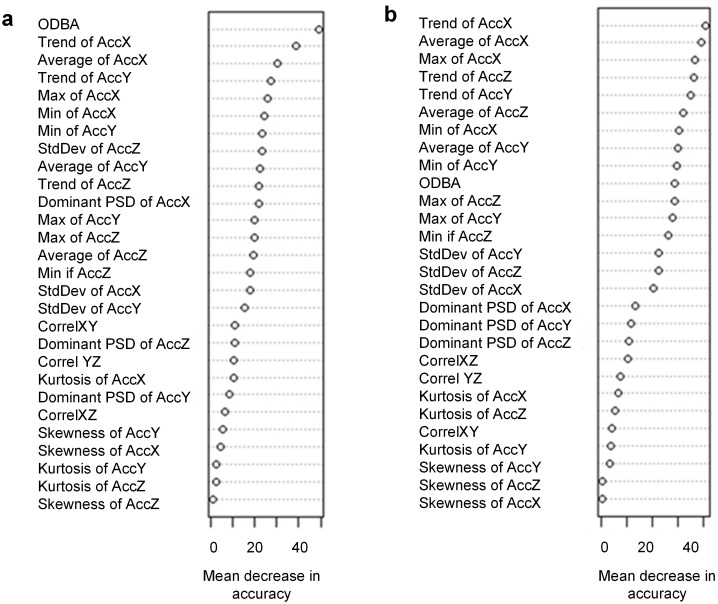
Variable importance plots for predictor variables (described in main text) from random forest classification of accelerometry data collected from a trained golden eagle. We classified data to (a) three behavioral classes: flapping, sitting and soaring and (b) five behavior classes: flapping banking, flapping straight, sitting, soaring banking and soaring straight. Higher values of mean decrease in accuracy indicate that the variables are more important to the classification process.

The RF model showed optimum performance with values of ntree and mtry at 1500 and 7, respectively, for the simple ethogram and 1000 and 7 for the complex ethogram (Fig 1a and 1b). For the simple ethogram, the most important variables were ODBA, trend of the *x* axis and average of the *x* axis (Fig 2a). For the complex ethogram, trend of the x axis, average of the *x* axis and the maximum of the *x* axis were the three most important variables based on mean decrease in accuracy (Fig 2b). In general, skewness and kurtosis were the least important variables (Fig 2a and 2b). The range, mean and standard deviation of all 28 variables are listed in S2 Table.

The optimal RF model was highly accurate in classifying the simple flight behaviors in the testing data set, including flapping (balanced accuracy = 85.5%), soaring (92.8%) and sitting (84.1%) with an overall accuracy of 86.6% (Kappa = 0.7482; Table 2, Table A in S3 Table). However when we applied the model to the complex classification scheme, overall model accuracy was low (61.64%; Kappa = 0.4733; Table 2, Table B in S3 Table). In both cases, prediction of sitting or perching behavior was highly accurate (above 82%).

**Table 2:** Model accuracy parameters for supervised classification of accelerometer data collected from a trained golden eagle. Parameters reported are sensitivity, specificity, positive predicted value, negative predicted value, prevalence and balanced accuracy as estimated for random forest (RF) model and K-nearest neighbor (KNN) models.

Model	Ethogram		Sensitivity	Specificity	Pos. Pred. Value	Neg. Pred. Value	Prevalence	Balanced Accuracy
RF	Simple	Flapping	0.8288	0.8810	0.6866	0.9424	0.2392	0.8549
		Sitting	0.7091	0.9731	0.7800	0.9614	0.1185	0.8411
		Soaring	0.9094	0.9458	0.9679	0.8533	0.6422	0.9276
	Complex	Flapping banking	0.7567	0.9414	0.5283	0.9781	0.0797	0.8491
		Flapping straight	0.6610	0.8938	0.4756	0.9476	0.1272	0.7774
		Sitting	0.6429	0.9924	0.9375	0.9399	0.1509	0.8176
		Soaring banking	0.6414	0.8451	0.8299	0.6667	0.5409	0.7432
		Soaring straight	0.2765	0.8225	0.1494	0.9098	0.1013	0.5496
KNN	Simple	Flapping	0.7395	0.9318	0.1363	0.9959	0.0143	0.8357
		Sitting	0.7837	0.9943	0.6287	0.9974	0.0121	0.8890
		Soaring	0.9270	0.8251	0.9949	0.2347	0.9736	0.8760
	Complex	Flapping banking	0.6800	0.9447	0.0653	0.9980	0.0056	0.8123
		Flapping straight	0.6784	0.9860	0.2757	0.9974	0.0078	0.8322
		Sitting	0.7664	0.9947	0.6584	0.9969	0.0132	0.8805
		Soaring banking	0.9197	0.8339	0.9950	0.2215	0.9728	0.8759
		Soaring straight	0.5000	0.9911	0.0304	0.9997	0.0006	0.7455

The estimates of error produced by the 10-fold validation process, 12.9% for the simple ethogram and 35.3% for the complex ethogram, were comparable to the errors estimated using the 70/30 split (Table 3).

**Table 3:** Ten-fold cross validation error rates for classification of accelerometer data collected from a trained golden eagle. Models tested were random forest (RF) and K-nearest neighbor (KNN).

Model	Ethogram	Minimum	1st Quartile	Median	Mean	3rd Quartile	Maximum
RF	Simple	12.91%	13.04%	13.17%	13.23%	13.39%	13.74%
	Complex	35.32%	35.90%	36.26%	36.13%	36.53%	36.67%
KNN	Simple	8.54%	8.55%	8.56%	8.57%	8.58%	8.59%
	Complex	9.78%	9.83%	9.85%	9.85%	9.89%	9.92%

#### K- nearest neighbor model

**Fig 3:**
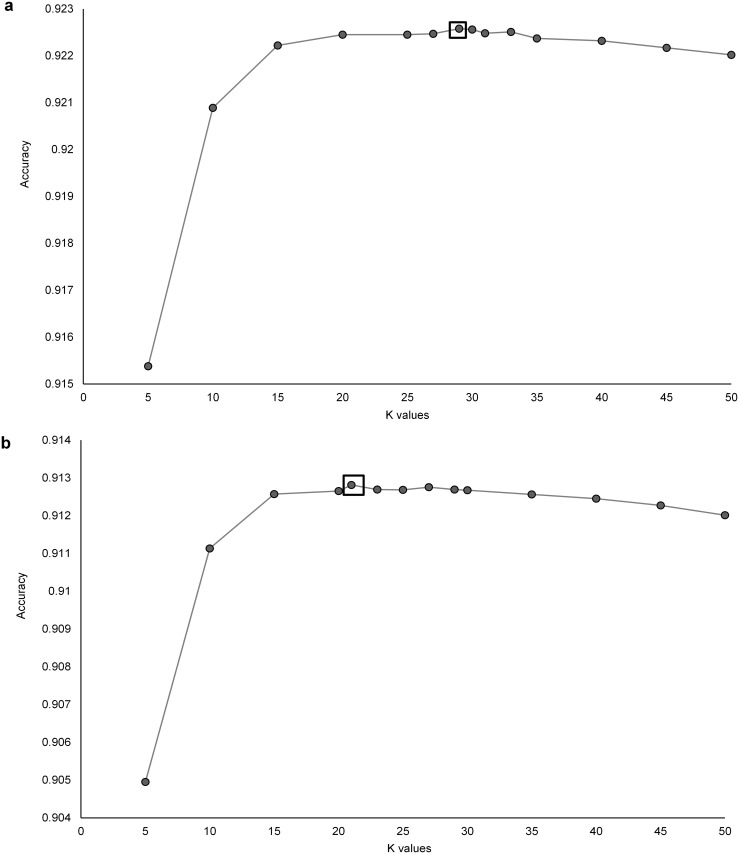
Overall classification accuracy using a K-nearest neighbor model to classify acceleration data from a trained golden eagle. We used both (a) a simple ethogram (three behavioral classes: flapping, sitting and soaring) and (b) a complex ethogram (five behavior classes: flapping banking, flapping straight, sitting, soaring banking and soaring straight). We incrementally increased values of K by 5 until classification accuracy declined and then incrementally adjusted values of K by 1 to identify peak accuracy, indicated with a box.

For the K-nearest neighbor model, we used 435,414 data points for training (70% of the data set) and 186,606 data points for testing (30% of the data set). The K-nearest neighbor model performed best at K = 29 for classification with the simple ethogram (Fig 3a) and K = 21 for the complex ethogram (Fig 3b). The model accurately classified both simple behaviors with classification of every class >83% accurate (92.25% overall accuracy, Kappa = 0.3299, Table 2, Table A in S4 Table) and more complex behaviors with classification of every class > 74.5% (91.24% overall accuracy, Kappa = 0.307, Table 2, Table B in S4 Table). As was the case with the RF model, the KNN model was most accurate at predicting sitting (88%).

The 10-fold validation process error estimates were again comparable to the errors estimated using the 70/30 split, at 8.54% for the simple ethogram and 9.78% for the complex ethogram (Table 3).

### Identifying ideal sampling frequencies for classification of accelerometry data

**Fig 4:**
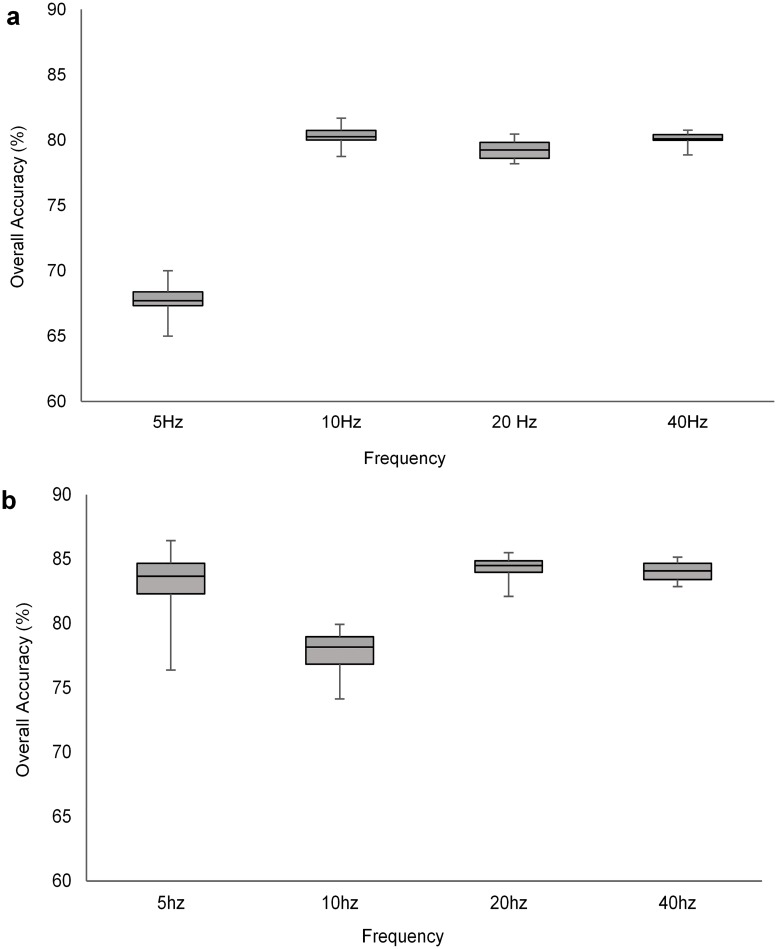
Accuracy of behavioral classification accuracy when sampling acceleration data from a trained golden eagle from 5 to 40Hz. Data were classified to three behavioral classes (flapping, sitting and soaring) and modeled with (a) a random forest classification model and (b) a K-nearest neighbor model.

Subsampling and re-analyses of 140 Hz accelerometer data suggested that the two models performed differently with when we subsampled those data. Ultimately, behavioral classification with the simple ethogram did not improve at sampling frequencies above 10 Hz for the RF model (Fig 4a) or above 20 Hz for the KNN model (Fig 4b).

### Application: Classification of data from wild eagles

**Fig 5:**
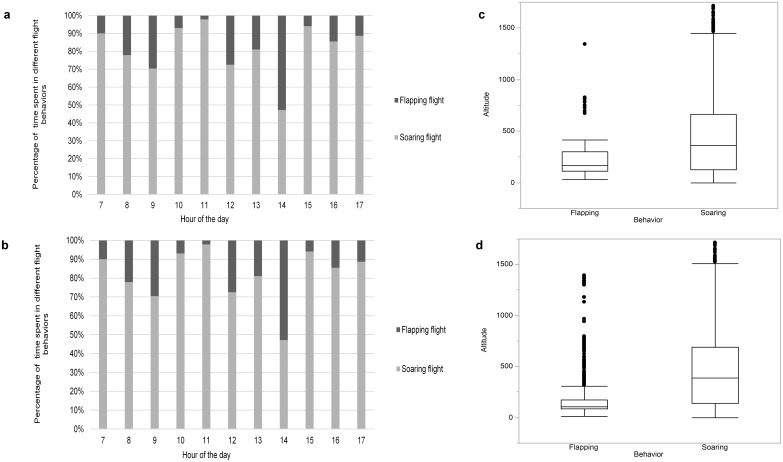
Flight behavior of 5 free-ranging golden eagles interpreted from acceleration data. Plots show percentage of time spent in flapping or soaring flight at different times of a day (a,b) and flight behavior as a function of flight altitude (c,d). Behavior was classified with (a, c) a random forest model and (b, d) a K-nearest neighbor model.

Results of classification of unvalidated data from wild eagles varied substantially depending on the classification algorithm used. The RF model suggested that the birds spent a total of 3.45% of the time in flapping flight and 96.55% of the time in soaring flight. In contrast, the KNN algorithm suggested that the birds spent 14.22% of the time in flapping flight and 85.78% of the time in soaring flight. As expected since the accelerometer only collected data in flight, the algorithm identified no behaviors as sitting or perching. For each of the five birds, the proportion of time spent flapping was time- and altitude-dependent (Fig 5).

## Discussion

Our analyses demonstrate the utility of accelerometry data to classify flight behavior, the consequence of different approaches to classification of accelerometry data, the potential to optimize classification algorithms with validated flight behaviors, and ideal sampling frequencies for different classification algorithms. Furthermore, we illustrate a number of ways to advance commonly used analytical techniques and that may form the basis for best practices for classification of accelerometry data.

### Using accelerometry data

Accelerometers are increasingly being used to quantify animal behavior and the dynamics of movement [29,30,41]. However processing and interpreting the multiple channels of accelerometer data collected 1–140 times/second presents considerable challenges [42]. Although techniques for processing acceleration data are being updated constantly [9,14,18,30] there are no fixed protocols that ensure the high classification accuracy of behaviors or that accelerometer data are calibrated to ground-truthed observations of tagged animals.

Earlier methodological studies have demonstrated the significance of different statistical approaches to interpreting accelerometer data [9] and of different approaches to segmenting those data [14]. Our work expands on those by comparing performance of an additional, often easier to implement, statistical model (the KNN) and illustrates that these different models may perform differently depending on the rate at which accelerometer data are collected.

A key component of our analysis was the use of detailed video recording of the bird for model training and validation. Field studies with accelerometry rarely train their model based on known behaviors and most provide no external validation of model outputs (e.g., [7,8,29,30]). As a consequence, those studies do not allow the reader to assess error rates in classification or interpretation; this is an important novel component to our work. Although the duration of our recordings was only 3 hours, we created a far larger validation data set than nearly all other similar accelerometer-based behavioral studies (see [9, 14] for studies with large validation datasets) and we found that our dataset was large enough to train our models to perform with high classification accuracies.

The use of combined metrics such as ODBA illustrates the pitfalls of interpreting accelerometry data without validation. ODBA derived from accelerometry is a commonly used metric to calculate energy expenditure [43–46]. However in our dataset ODBA was only sometimes useful in characterizing the difference between soaring and flapping flight (Fig 2) and this metric was highly variable, regardless of behavior (S1 Fig). Thus the use of models of energetic expenditure based solely on ODBA should be interpreted with care and this issue deserves further more detailed study.

### Optimizing analytical models and best practices

Although recent analytical papers using accelerometry have detailed the application and efficiency of different classification algorithms, they do not detail steps to optimize these models. We identified three critical components to the optimization process.

The first critical component was optimizing the parameters of the algorithm used for classification. For the RF models, there are a set of suggested default values that are generally used in published studies. However, as was expected [32,47], the accuracy of behavior classification improved significantly when we moved away from those mtry and ntree defaults. Likewise, for the KNN models, although the square root of the number of observations is often used for K, optimizing K improved our model classification. Although there is no consensus on the best way to pick K values, our approach (detailed in Methods) was an efficient and apparently effective solution to this problem.

The second component of the optimization process was the use of variable time segments to partition our accelerometer data and annotate behaviors. Although we did not test the effect of different types of segmentation on classification performance, variable time segments are generally preferred when acceleration data are collected at frequencies > 20 Hz and when the species under study shows short phases of dynamic behavior [14]. Our accelerometry data met these criteria because they were collected at 140 Hz on eagles that rapidly transition from flapping to soaring flight. A fixed-time segmentation would have been unlikely to pick up these quick transitions. Furthermore, we suspect that fixed-time segmentation likely also would be less effective for small species that move more rapidly than the large eagle we studied.

The third component of optimization involved characterizing ideal sampling frequencies for data collection. This is especially important for accelerometry data collected via remote telemetry systems on free-ranging animals because there are often memory and network bandwidth-related limitations to data storage and transmission. In fact, our experimental data collection on eastern eagles was, to our knowledge, the first time when accelerometry data from wildlife were transmitted over the mobile phone network. A general guideline in signal processing of accelerometer data is that the sampling frequency should be at least twice that of the highest frequency movement being classified [15,48]. The most common sampling frequencies used in wildlife studies have been at 10, 16 and 32 Hz [15]. However, it is unusual for authors to provide a rationale for choosing a particular sampling frequency. Our work shows that optimization of a sampling frequency depends strongly on the classification algorithm that analysts use. The 20 Hz sampling threshold we identified for KNN interpretation of data from eagles is likely appropriate for similarly sized or larger birds but may be inadequate to accurately classify behavior of smaller, faster moving, animals.

### Model application and limitations

The free ranging eagles we studied were soaring the majority of their time in flight. However, our analysis still provided evidence of diel cycles and altitude-specific patterns in behavior (Fig 5). These general patterns are consistent with other estimates of eagle behavior [12], and they could be comprehensively evaluated with a more robust dataset and statistical model that controlled for individual- and region-specific differences. As data transmission and processing capacities grow, accelerometry data ultimately may be paired with magnetometer and barometric data to further classify behavior of free ranging animals. To date similar studies have only been conducted with data loggers (as opposed to transmitters; [11]).

Our analysis also demonstrates potential limitations to accelerometry studies linked to classification of complex flight categories. Both the RF and KNN models were highly accurate in classifying the simple ethogram consisting of basic movement categories such as flapping, sitting and soaring. However, complex flight behaviors were poorly differentiated with the RF model. Application of these models to free-ranging eagles also demonstrated that these among-model differences subsequently resulted in substantial differences in behavior as estimated by the two models. Such model-dependent differences in performance would have substantial implications for the many published studies that have not attempted to validate flight behavior. Resolution of these differences and continued optimization of these models is therefore likely important to further understanding of animal behavior.

## Conclusion

Accelometry provides ecologists a tool to monitor detailed changes in behavior of free-ranging animals. However, collection and use of accelerometry data are challenging because existing protocols are relatively new and not highly refined, and the entire process presents a series of logistical and computational challenges. The protocols we provide here allow species- and model-specific optimization via ground-truthed data that would improve confidence in behavioral, and ultimately energetic, classification from accelerometer data. This will allow further understanding of species-specific behavioral mechanisms and energetics of free ranging wild animals and, furthermore, standardize analytics for inter-specific comparisons that can inform as to the evolution and function of specific behaviors.

## Supporting information

S1 Supporting Information Supplemental methods. (PDF)

S2 Supporting Information Definition of statistics calculated from confusion matrices generated by models for classification of accelerometer data. (PDF)

S1 Table Amount of video collected from a trained golden eagle outfitted with an accelerometer. (PDF)

S2 Table Descriptive statistics for the 28 variables calculated for the segments defined by the change point model. (PDF)

S3 Table Confusion matrix of RF classification predictions. (PDF)

S4 Table Confusion matrix of KNN classification predictions. (PDF)

S1 Fig Box plot of Overall Dynamic Body Acceleration (ODBA). (PDF)
