# Supplementary material for: Improved supervised classification of accelerometry data to distinguish behaviors of soaring birds
Source: PLoS One. 2017 Apr 12;12(4):e0174785. doi: 10.1371/journal.pone.0174785 (PMC5389810; doi:10.1371/journal.pone.0174785)
Supplement: S1 Supporting Information — (PDF) [file pone.0174785.s001.pdf]

S1 Supporting Information. Supplemental methods

We flew the trained golden eagle at three sites in southern California. The first, in the foothills near the town of Tehachapi, CA, (hereafter referred to as "Tehachapi") is scrub-oak savanna forest, dominated by annual and perennial grasses and a variety of mid-elevation California oaks (*Quercus spp*.) and pines (*Pinus spp.*). Golden eagles occur in high densities in this region, both as breeders and non-breeders, and they feed especially on California ground squirrels (*Otospermophilus beecheyi*; [1,2]). At this site we (a) induced low-altitude soaring and occasional flapping flight by releasing the eagle from above a cliff while recording its flight in response to the local topography and (b) simulated hunting behavior by recording the eagle as it engaged in rapid flapping flight to chase and grab a lure dragging behind a toy remote control car.

The second site was disturbed greasewood scrub habitat in a flat plain of low desert habitat near the town of Arvin, CA (hereafter "scrub"). Golden eagles are occasional visitors to this habitat type, although there are few records of eagles breeding in the immediate vicinity. Here we carried the bird through the field and allowed it to hunt rabbits that our walking disturbed (i.e., we flew the bird as a typical falconer would). Data from this site replicate some of the hunting behaviors of low flying eagles.

The third site was Mojave Desert scrub near the town of Apple Valley, CA, (hereafter "Mojave"). Vegetation at this site was dominated by creosote bush (*Larrea tridentate*), Joshua trees (*Yucca brevifolia*) and other typical desert plants [3,4]. Here again, we released the eagle from a hilltop and recorded it flying in response to local topography. At this site the eagle flew higher and farther than it did at any other site, at one point travelling out of view for nearly 30 minutes.

References

1. Carnie, S.K. (1954) Food habits of nesting golden eagles in the coast ranges of California. The Condor, 56(1), 3-12.
2. Poessel, S.A., Bloom, P.H., Braham, M.A. and Katzner, T.E. (2016) Age-and season-specific variation in local and long-distance movement behavior of golden eagles. European Journal of Wildlife Research, 1-17.
3. Brown, D.E. (1994) Biotic communities: southwestern United States and northwestern Mexico. University of Utah Press, Sat Lake City, USA.
4. Braham, M., Miller, T., Duerr, A.E., Lanzone, M., Fesnock, A., LaPre, L., Driscoll, D. and Katzner, T. (2015) Home in the heat: Dramatic seasonal variation in home range of desert golden eagles informs management for renewable energy development. Biological Conservation, 186, 225-232.
