# Supplementary material for: Improved supervised classification of accelerometry data to distinguish behaviors of soaring birds
Source: PLoS One. 2017 Apr 12;12(4):e0174785. doi: 10.1371/journal.pone.0174785 (PMC5389810; doi:10.1371/journal.pone.0174785)
Supplement: S2 Supporting Information — (PDF) [file pone.0174785.s002.pdf]

**S2 Supporting Information. Definition of statistics calculated from confusion matrices generated by models for classification of accelerometer data**. See text for details on data collected and other methods.

*Acronyms:*

TP= True positive

TN= True negative

FP= False positive

FN= False negative

**Accuracy:** Overall, describes frequency with which the classifier is correct.

*Accuracy = (TP+TN)/ (TP+FP+FN+TN)*

**Kappa:** A measure of how well the classifier performed as compared to how well it would have performed simply by chance.

**P value:** A weighted average of the true positive rate (recall) and precision.

**Sensitivity or true positive rate (TPR):**

The proportion of occurrences of a behavior that are accurately predicted (i.e., the model predicted a behavior and it actually occurred).

*TPR=TP/ (TP+FN)*

**Specificity (SPC) or true negative rate:**

The proportion of occurrences when a behavior does not occur that are accurately predicted (i.e., the model predicted the behavior did not occur and it did not).

*SPC- TN/ (TN+FP)*

**Precision or positive predictive value (PPV):**

The proportion of predictions of a behavior that are accurate (i.e., the behavior actually occurred when the model predicted it did).

$PPV = TP/ (TP+FP)$

**Negative predictive value (NPV):**

The proportion of predictions that a behavior does not occur that are accurate (i.e., the behavior did not occur but the model predicted it did).

$NPV = TN/ (TN+FN)$

**Prevalence (PRV):**

The proportion of correctly predicted occurrences composed of a single behavior.

$PRV = TP/ (TP+FP+FN+TN)$

**Balanced Accuracy (BA):**

The mean of sensitivity and specificity by behavior type

$BA = (Sensitivity+Specificity)/2$
