# Supplementary material for: Improved supervised classification of accelerometry data to distinguish behaviors of soaring birds
Source: PLoS One. 2017 Apr 12;12(4):e0174785. doi: 10.1371/journal.pone.0174785 (PMC5389810; doi:10.1371/journal.pone.0174785)
Supplement: S1 Table — (PDF) [file pone.0174785.s003.pdf]

S1 Table. Amount of video collected from a trained golden eagle outfitted with an accelerometer. Although video was collected in continuous segments at each site, the bird was only in the frame ("visible" below) for a subset of the time the camera was running. Bird was also perched on the trainer's hand ("handling") at times during recording sessions. See Methods for details on accelerometry and video data collection.

Location	Time recorded	Time bird not visible	Total time bird visible	Total flying/sitting time (minus handling time)
Tehachapi cliff	0:13:44	0:07:03	0:06:41	0:05:18
Tehachapi ranch	0:01:46	0:00:00	0:01:46	0:01:05
Scrub	0:18:04	0:00:00	0:18:04	0:02:12
Mojave	0:29:35	0:25:45	0:03:50	0:06:35
**Total**	**1:03:09**		**0:30:21**	**0:15:10**
