# Supplementary material for: Improved supervised classification of accelerometry data to distinguish behaviors of soaring birds
Source: PLoS One. 2017 Apr 12;12(4):e0174785. doi: 10.1371/journal.pone.0174785 (PMC5389810; doi:10.1371/journal.pone.0174785)
Supplement: S2 Table — (PDF) [file pone.0174785.s004.pdf]

S2 Table. **Descriptive statistics for the 28 variables calculated for the segments defined by the change point model.** Descriptors are reported for each axis and for four multi-axis parameters. Dominant PSD =Dominant power spectral density, CorrelXY = correlation between X and Y axes, CorrelYZ = correlation between Y and Z axes, CorrelXZ = correlation between X and Z axes and ODBA = overall dynamic body acceleration.

		**Mean**	**Min**	**Max**	**Std. Dev.**	**Skewness**	**Kurtosis**	**Trend**	**DominantPSD**
**X axis**	Min	-6.5894	-5.2377	-20.9219	0.0048	-3.1661	1.0000	-6.6255	0.0553
	Max	16.7119	33.5967	7.8748	12.3722	3.1266	20.5682	8.1902	887898.9859
	Mean	0.3012	1.5934	-1.1008	0.6773	-0.0365	2.7838	0.2686	29075.1163
	StdDev	2.8186	3.0049	3.9520	0.9992	0.6121	1.3139	2.7975	69037.4632
**Y axis**	Min	-6.6778	-6.5492	-18.2282	0.0031	-2.3075	1.0000	-6.7276	3.4686
	Max	12.4557	39.7790	10.7873	10.5190	2.3215	9.9980	10.5678	902417.0935
	Mean	3.4352	5.3138	1.8160	0.9260	0.0909	2.6275	3.4382	69440.9429
	StdDev	2.7110	3.9163	3.2294	1.2259	0.6027	1.0159	2.6348	105421.7147
**Z axis**	Min	-22.3660	-18.5529	-49.5028	0.0029	-2.3345	1.0000	-23.2783	13.1413
	Max	8.1695	20.2678	6.2321	14.8786	2.2191	10.1071	-0.6818	6946621.4190
	Mean	-9.6104	-7.1018	-12.1690	1.3245	-0.0212	2.7025	-9.6743	449747.5560
	StdDev	2.9506	4.8481	4.7032	1.9485	0.5984	1.0856	2.9460	709374.3798

	**CorrelXY**	**CorrelYZ**	**CorrelXZ**	**ODBA**
**Min**	-1	-1	-1	0.1252
**Max**	1	1	1	72.9441
**Mean**	-0.03713	-0.4032	0.1633	3.6156
**StdDev**	0.4404	0.3884	0.4526	5.4394
