# Supplementary material for: Improved supervised classification of accelerometry data to distinguish behaviors of soaring birds
Source: PLoS One. 2017 Apr 12;12(4):e0174785. doi: 10.1371/journal.pone.0174785 (PMC5389810; doi:10.1371/journal.pone.0174785)
Supplement: S3 Table — (PDF) [file pone.0174785.s005.pdf]

S3 Table. **Confusion matrix of RF classification predictions.** Predictions were made on 30% (464 segments) of accelerometer data collected from a trained golden eagle and classified with a random forest model and (A) a simple ethogram (three behavioral classes: flapping, sitting and soaring) and (B) a complex ethogram (five behavior classes: flapping banking, flapping straight, sitting soaring banking and soaring straight).

(A)

	Flapping	Sitting	Soaring	**Total**
Flapping	92	16	26	**134**
Sitting	10	39	1	**50**
Soaring	9	0	271	**280**
**Total**	**111**	**55**	**298**	**464**

(B)

	Flapping banking	Flapping straight	Sitting	Soaring banking	Soaring straight	**Total**
Flapping banking	28	12	7	3	3	**53**
Flapping straight	6	39	17	15	5	**82**
Sitting	1	1	45	1	0	**48**
Soaring banking	1	6	0	161	26	**194**
Soaring straight	1	1	1	71	13	**87**
**Total**	**37**	**59**	**70**	**251**	**47**	**464**
