# Supplementary material for: Improved supervised classification of accelerometry data to distinguish behaviors of soaring birds
Source: PLoS One. 2017 Apr 12;12(4):e0174785. doi: 10.1371/journal.pone.0174785 (PMC5389810; doi:10.1371/journal.pone.0174785)
Supplement: S4 Table — (PDF) [file pone.0174785.s006.pdf]

S4 Table. **Confusion matrix of KNN classification predictions.** Predictions were made on 30% of accelerometer data collected from a trained golden eagle and classified with a K-nearest neighbor model and (A) a simple ethogram (three behavioral classes: flapping, sitting and soaring) and (B) a complex ethogram (five behavior classes: flapping banking, flapping straight, sitting soaring banking and soaring straight).

(A)

	Flapping	Sitting	Soaring	**Total**
Flapping	1979	186	12353	**14518**
Sitting	136	1765	906	**2807**
Soaring	561	301	168418	**169280**
**Total**	**2676**	**2252**	**181677**	**186605**

(B)

	Flapping Banking	Flapping Straight	Sitting	Soaring Banking	Soaring Straight	**Total**
Flapping Banking	716	136	102	10010	5	**10969**
Flapping Straight	99	987	140	2350	4	**3580**
Sitting	30	63	1893	887	2	**2875**
Soaring Banking	205	263	335	166626	41	**167470**
Soaring Straight	3	6	0	1651	52	**1712**
**Total**	**1053**	**1455**	**2470**	**181524**	**104**	**186606**
