# Supplementary material for: Improved supervised classification of accelerometry data to distinguish behaviors of soaring birds
Source: PLoS One. 2017 Apr 12;12(4):e0174785. doi: 10.1371/journal.pone.0174785 (PMC5389810; doi:10.1371/journal.pone.0174785)

S1 Fig. **Box plot of overall dynamic body acceleration (ODBA).** ODBA was calculated based on accelerometer data collected from a trained golden eagle engaged in three distinct behaviors (flapping, sitting and soaring) interpreted from video of the eagle in flight.

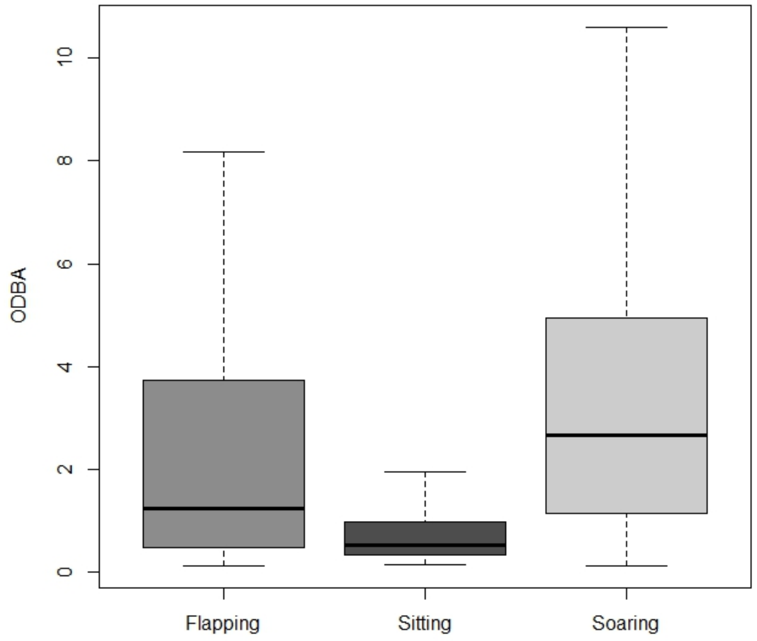

Supplement: S1 Fig — (PDF) [file pone.0174785.s007.pdf]
